# Supplementary material for: One-stone-for-two-birds strategy to attain beyond 25% perovskite solar cells
Source: Nat Commun. 2023 Feb 15;14:839. doi: 10.1038/s41467-023-36229-1 (PMC9932071; doi:10.1038/s41467-023-36229-1)
Supplement: Supplementary file 1 — Supplementary Information [file 41467_2023_36229_MOESM1_ESM.pdf]

Supplementary Information

One-stone-for-two-birds strategy to attain beyond 25% perovskite solar cells

Yang et al.

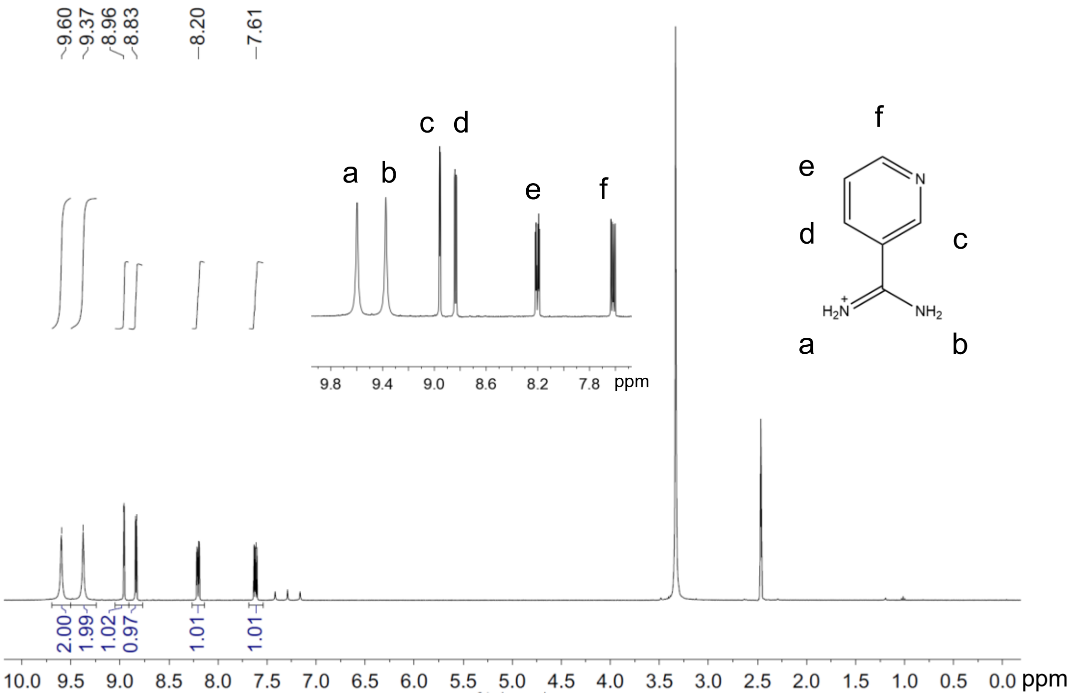

Supplementary Fig. 1| ^{1}H-NMR spectra of $3AP^+$ (d_6-DMSO, 400 MHz, ppm). a and b represent $=NH_2^+$ and $-NH_2$ of the amidino group in the molecular structure of $3AP^+$, respectively. c-f represent =CH– at different positions on the pyridine ring in $3AP^+$, respectively. a-f corresponding ^{1}H-NMR assignments are shown in the inset.

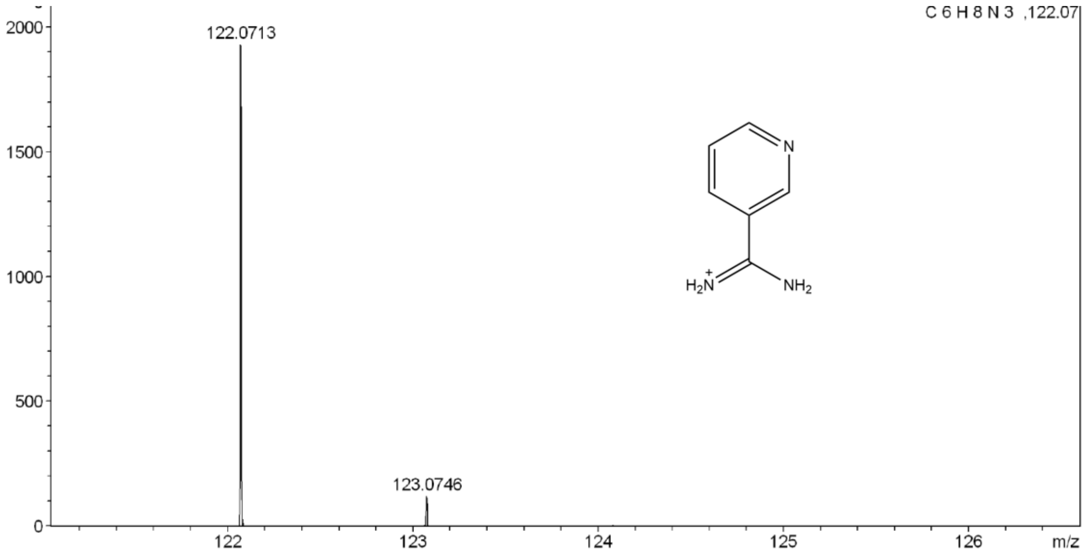

Supplementary Fig. 2| High-resolution mass spectral (HRMS) of $3AP^+$.

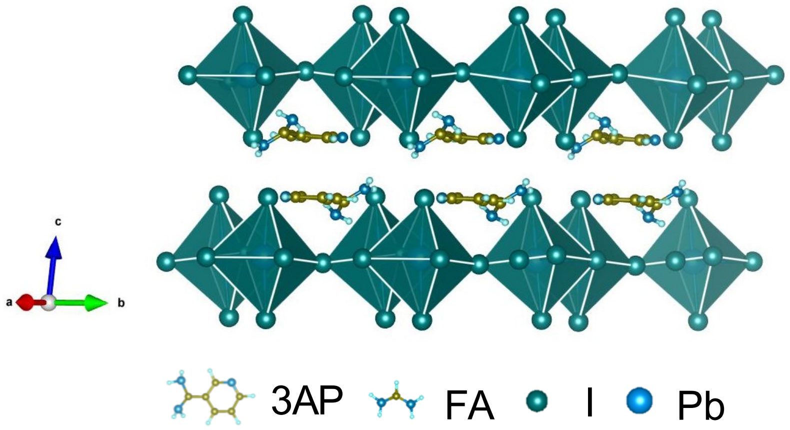

Supplementary Fig. 3| $(3AP)PbI_4$ structure.

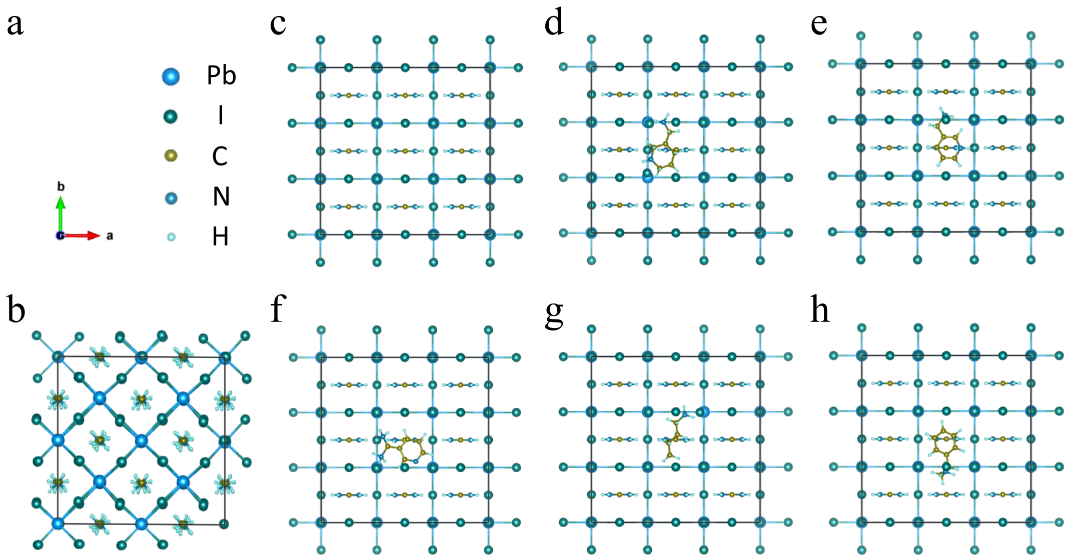

Supplementary Fig. 4| The crystal structure model constructed by the first-principles calculations in the frame of density functional theory (DFT) with the program package CASTEP. (a) Legend and crystal coordinates. (b) $MAPbI_3$-001. (c) $FAPbI_3$-001. (d-h) 3AMP, 3AMPY, 3AP, BA and PEA interact with $FAPbI_3$ terminals, respectively.

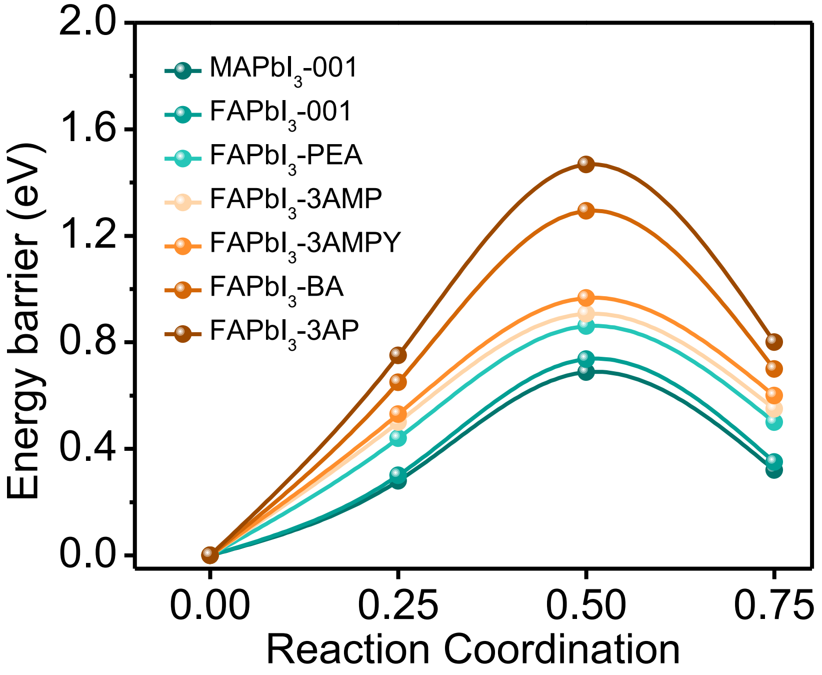

Supplementary Fig. 5| The energy barrier of I^- diffusion on FAI-terminal $FAPbI_3$ (001) surface and MAI-terminal $MAPbI_3$ (001) surface.

5

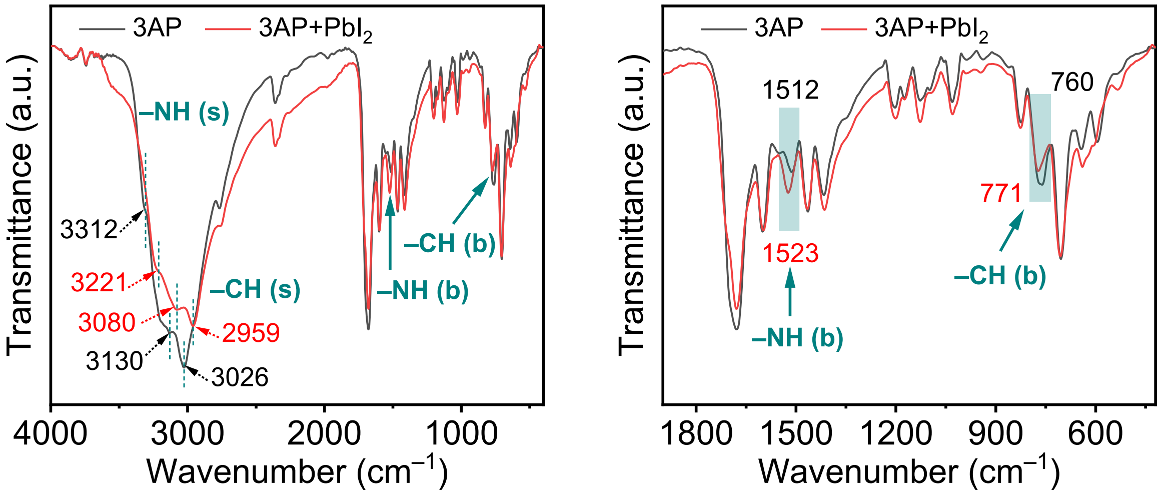

Supplementary Fig. 6| FTIR spectra of 3AP and $3AP+PbI_2$ compounds.

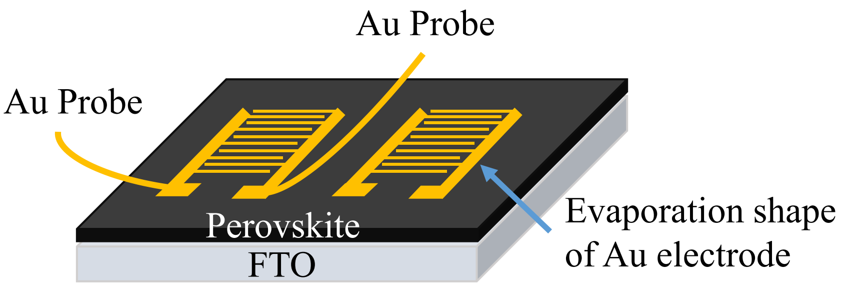

Supplementary Fig. 7| The device structure for the temperature-dependent conductivity measurements.

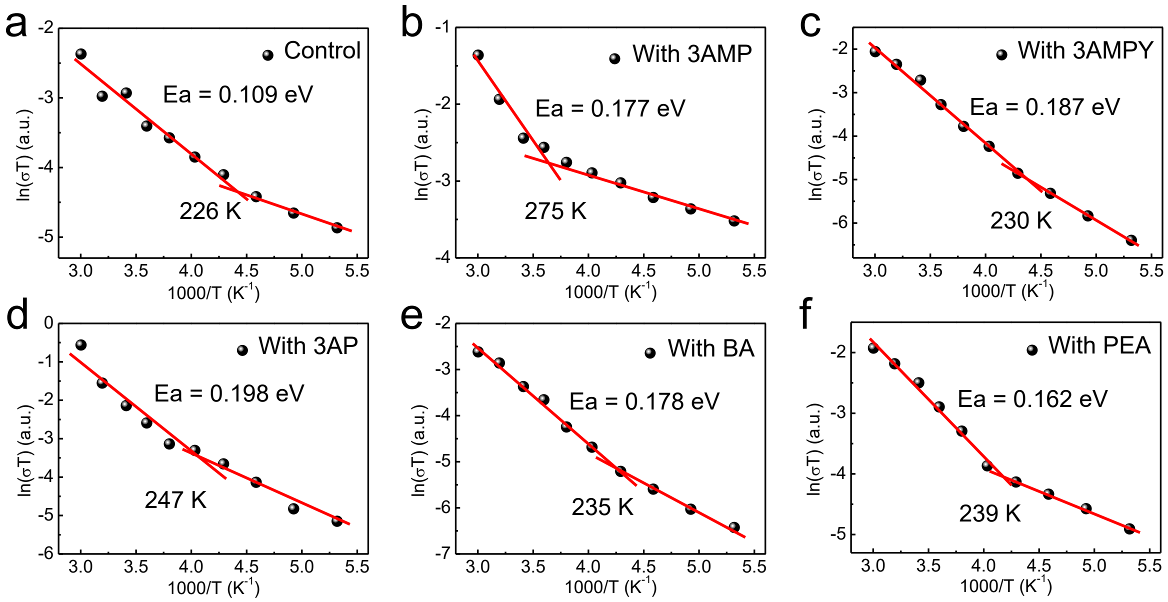

Supplementary Fig. 8| Temperature-dependent conductivities of the perovskite films without (a) ligands (denoted as control) and with (b) 3AMP, (c) 3AMPY, (d) 3AP, (e) BA, and (f) PEA ligands.

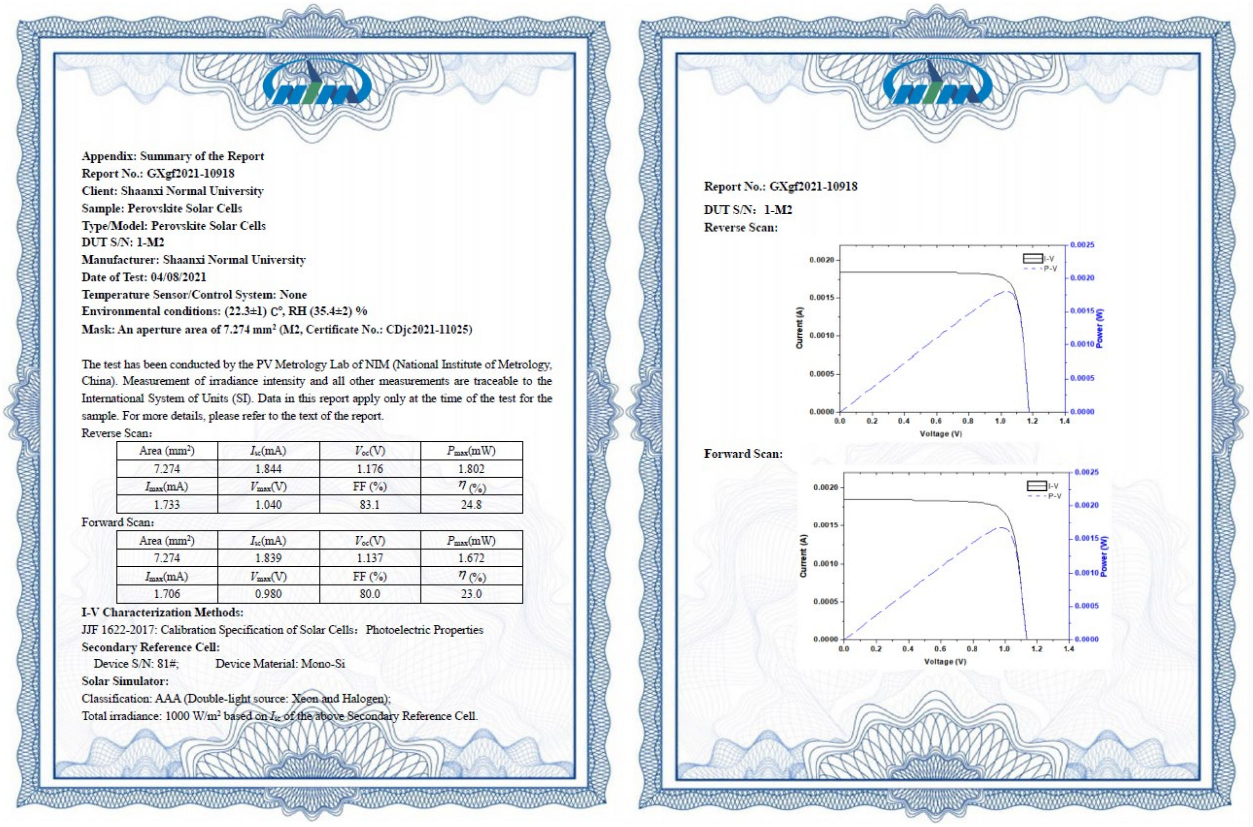

Appendix: Summary of the Report
Report No.: GXgf2021-10918
Client: Shaanxi Normal University
Sample: Perovskite Solar Cells
Type/Model: Perovskite Solar Cells
DUT S/N: 1-M2
Manufacturer: Shaanxi Normal University
Date of Test: 04/08/2021
Temperature Sensor/Control System: None
Environmental conditions: (22.3±1) C°, RH (35.4±2) %
Mask: An aperture area of 7.274 mm² (M2, Certificate No.: CDjc2021-11025)

The test has been conducted by the PV Metrology Lab of NIM (National Institute of Metrology, China). Measurement of irradiance intensity and all other measurements are traceable to the International System of Units (SI). Data in this report apply only at the time of the test for the sample. For more details, please refer to the text of the report.

Reverse Scan:

Area (mm^2)	I_{sc}(mA)	V_{oc}(V)	P_{max}(mW)
7.274	1.844	1.176	1.802
I_{max}(mA)	V_{max}(V)	FF (%)	η (%)
1.733	1.040	83.1	24.8

Forward Scan:

Area (mm^2)	I_{sc}(mA)	V_{oc}(V)	P_{max}(mW)
7.274	1.839	1.137	1.672
I_{max}(mA)	V_{max}(V)	FF (%)	η (%)
1.706	0.980	80.0	23.0

I-V Characterization Methods:
JJF 1622-2017: Calibration Specification of Solar Cells：Photoelectric Properties
Secondary Reference Cell:
Device S/N: 81#; Device Material: Mono-Si
Solar Simulator:
Classification: AAA (Double-light source: Xeon and Halogen);
Total irradiance: 1000 W/m² based on I_{sc} of the above Secondary Reference Cell.

Report No.: GXgf2021-10918
DUT S/N: 1-M2
Reverse Scan:

Forward Scan:

Supplementary Fig. 9| Certification of the PSCs with 3API at the National Institute of Metrology, China. The PCE is 24.8% (V_{OC} = 1.176 V, I_{SC} = 1.844 mA, FF = 83.1%) at the reverse scan. The PCE is 23.0% (V_{OC} = 1.137 V, I_{SC} = 1.839 mA, FF = 80.0%) at the forward scan. The aperture area of the device is 7.274 mm^2.

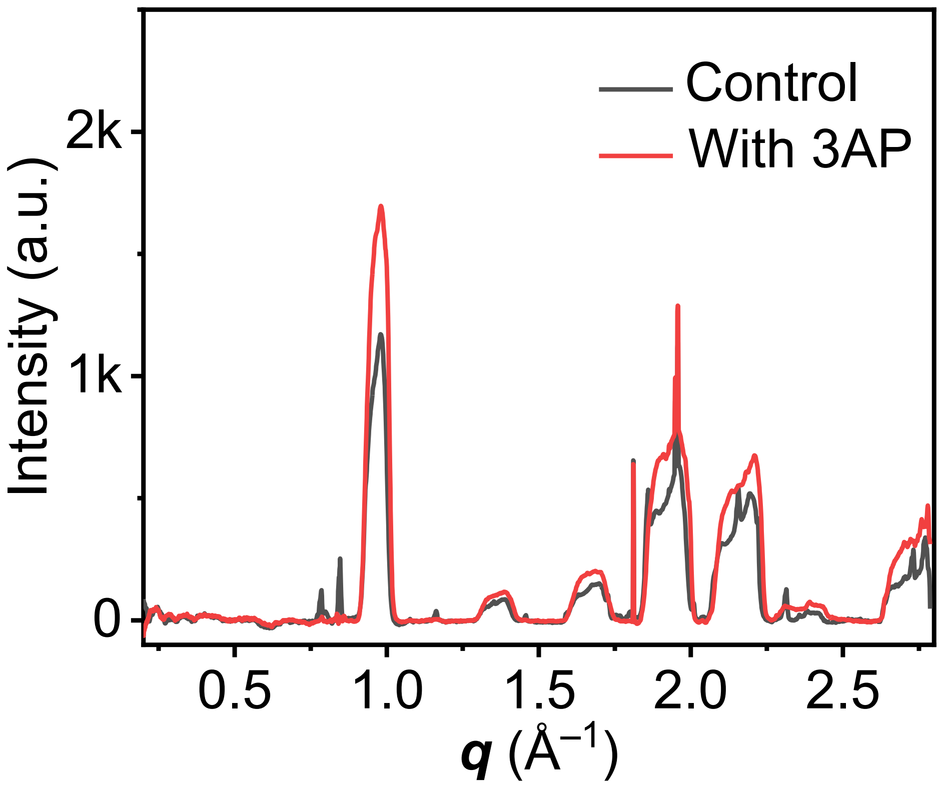

Supplementary Fig. 10| The line-plot file of GIWAXS of control and with 3AP films.

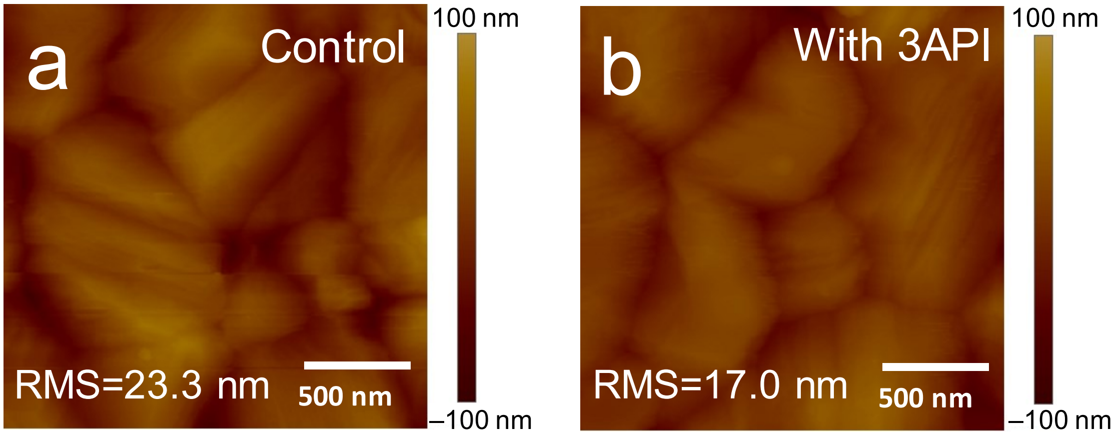

Supplementary Fig. 11| AFM images of the (a) control and (b) 3API-based perovskite films.

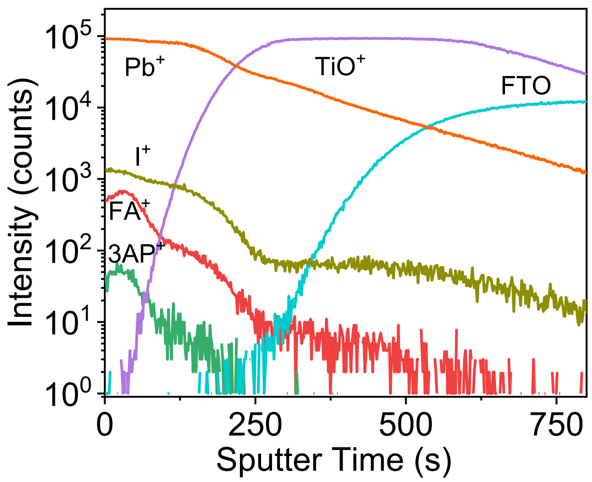

Supplementary Fig. 12| The characterization of TOF-SIMS for $FAPbI_3$ perovskite film with 3API.

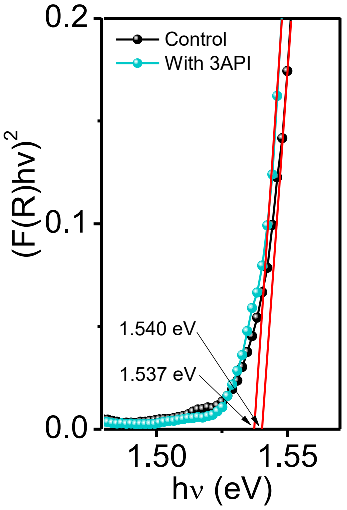

Supplementary Fig. 13| Tauc plots of the perovskite films with/without 3API.

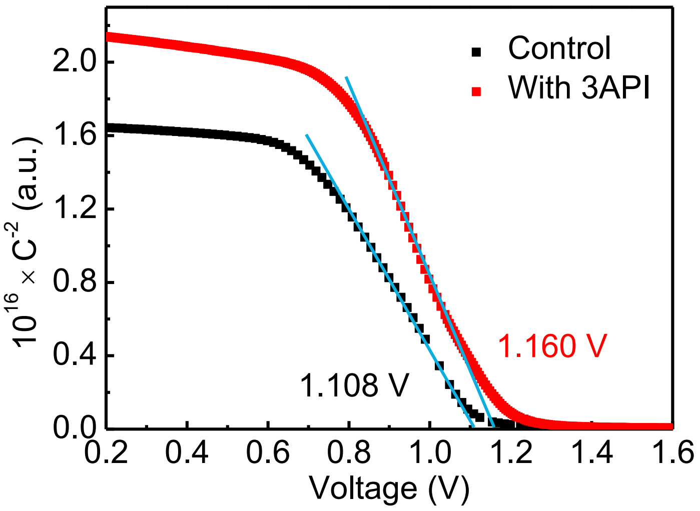

Supplementary Fig. 14| Mott–Schottky plots of the control and 3AP-based PSCs.

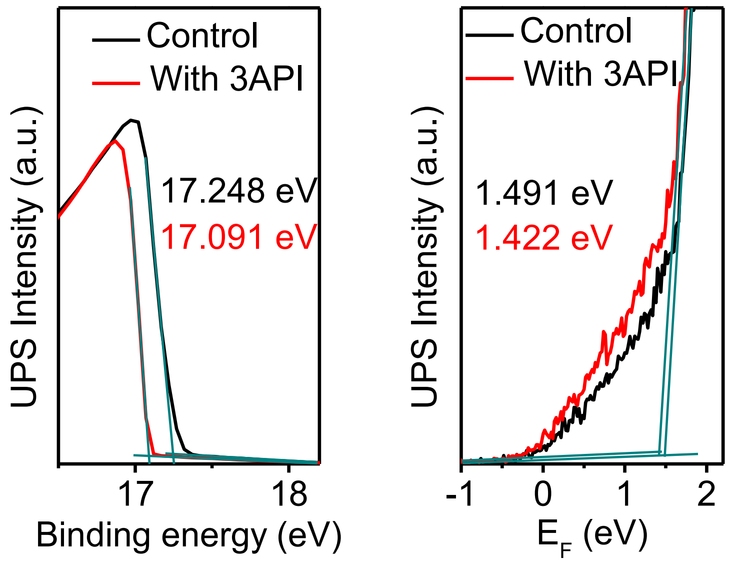

Supplementary Fig. 15| UPS measurements and band gaps of films with/without 3API.

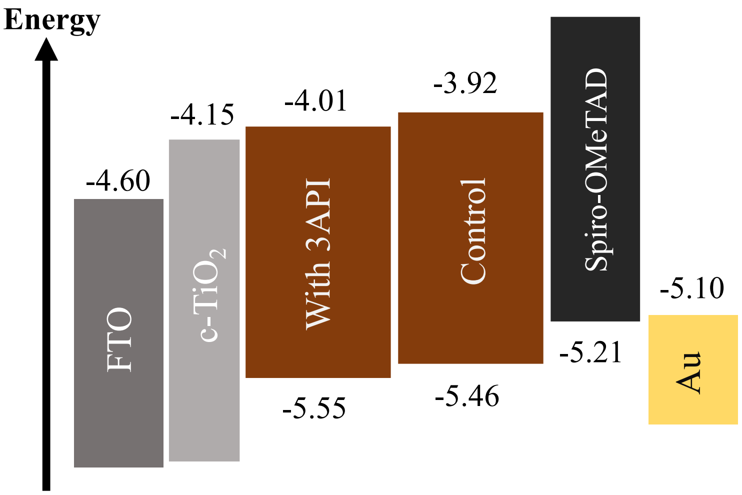

Supplementary Fig. 16| The energy level alignment of perovskite solar cells.

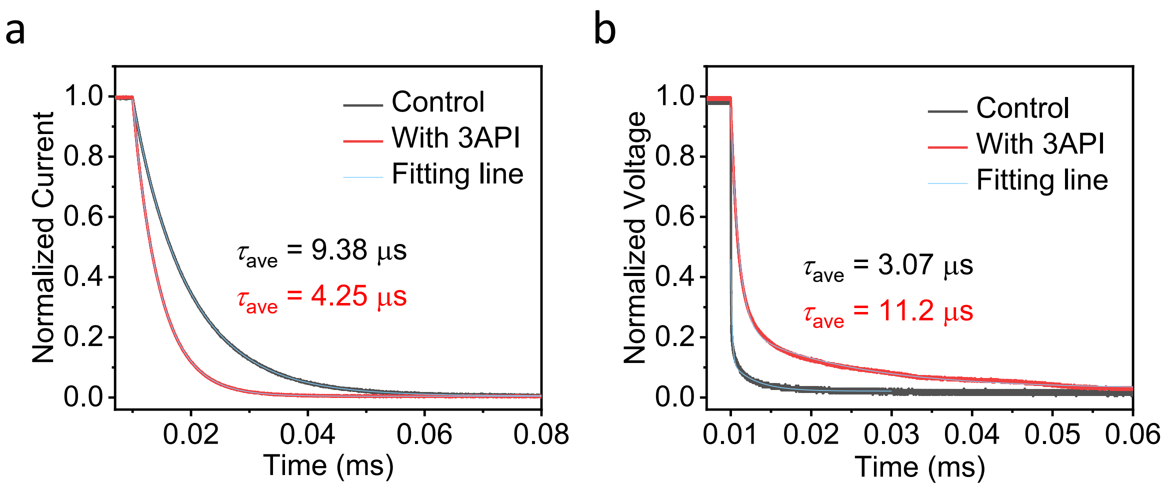

Supplementary Fig. 17| Transient photocurrent and photovoltage characterization. (a) Transient photocurrent (TPC) and (b) transient photovoltage (TPV) decay curves of PSCs for control and with 3API.

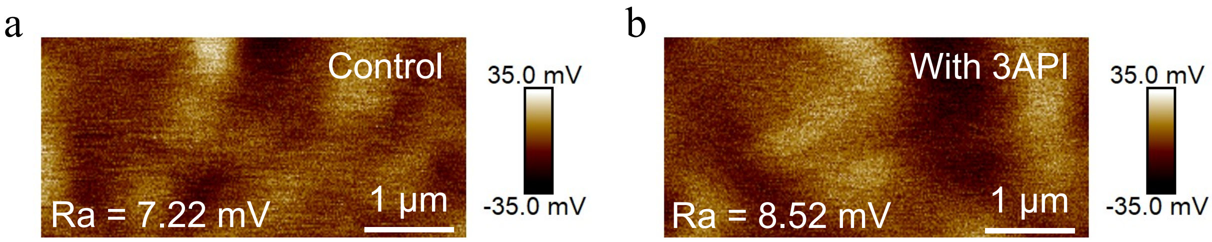

Supplementary Fig. 18| KPFM images of perovskite films for Control (a) and with 3API (b).

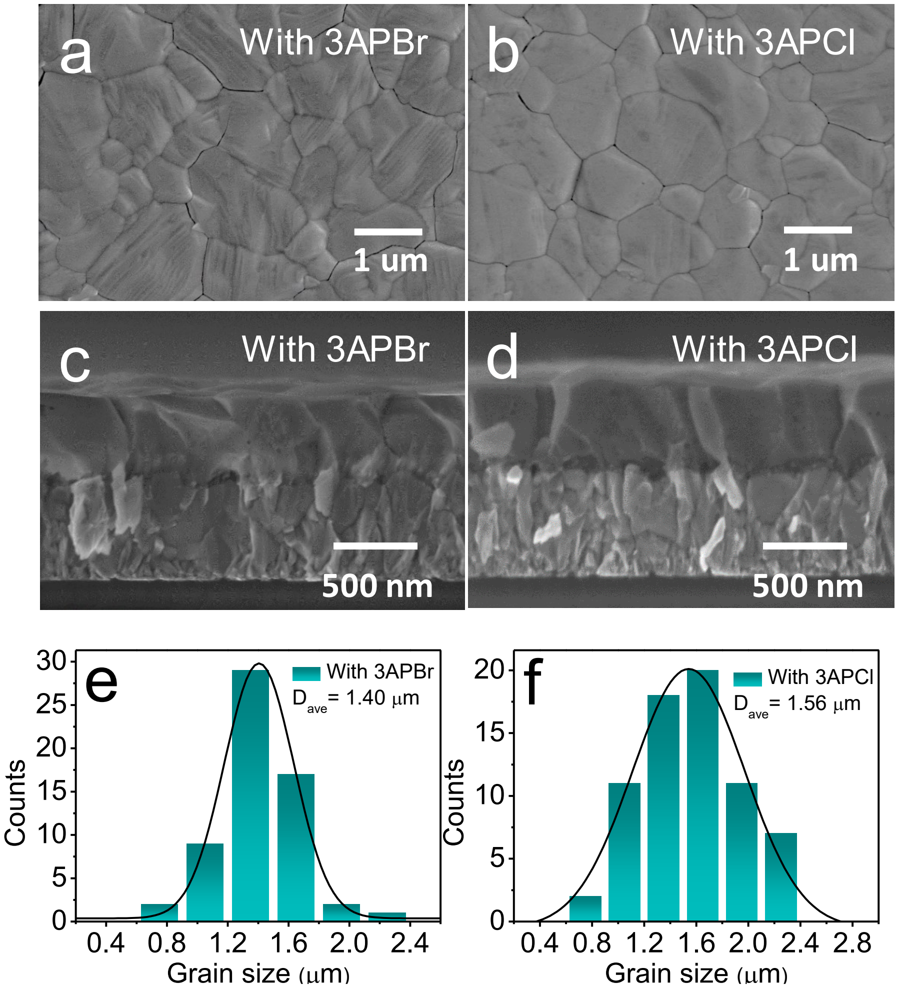

Supplementary Fig. 19| SEM images (a, b), cross-sectional SEM images (c, d) and the corresponding grain size distributions (e, f) of the perovskite films with 3APBr and 3APCl, respectively.

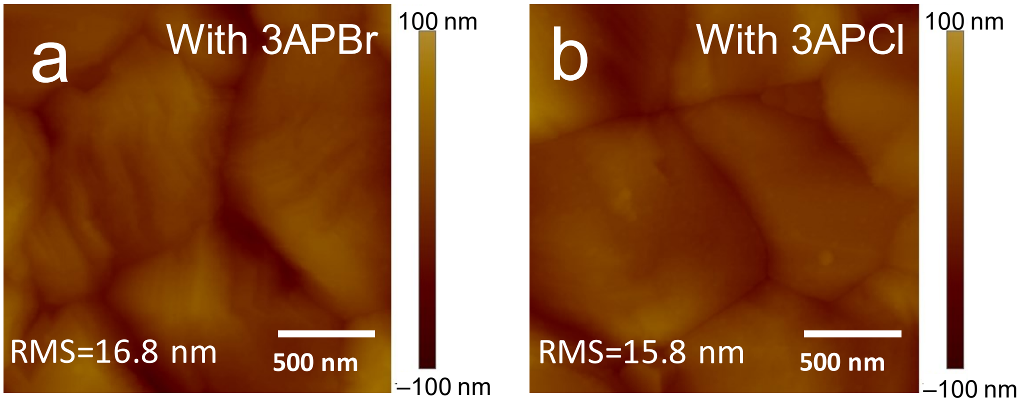

Supplementary Fig. 20| AFM images of the perovskite films with (a) 3APBr and (b) 3APCl.

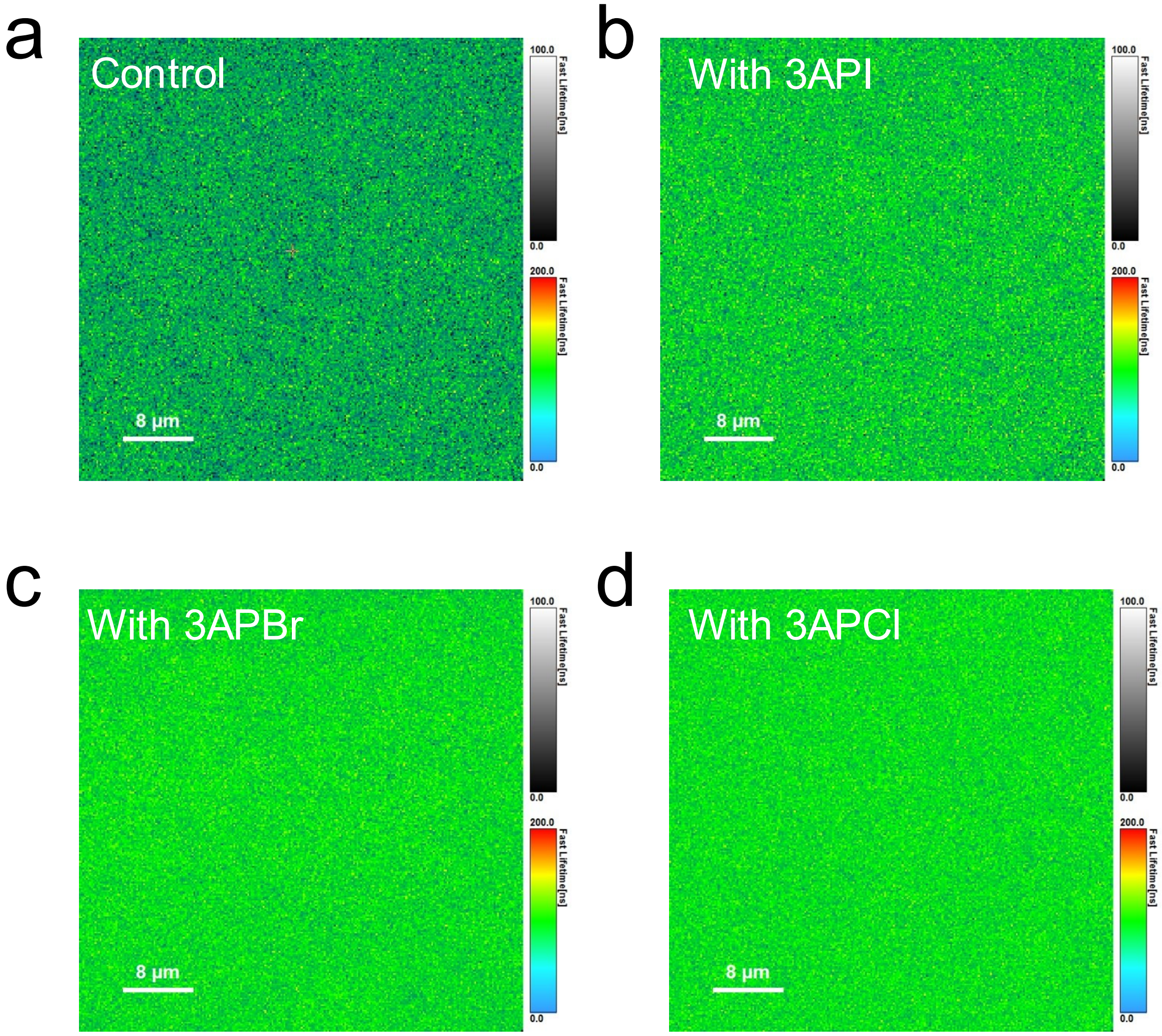

Supplementary Fig. 21| PL maps of the perovskite films. (a) without additives and with (b) 3API, (c) 3APBr, and (d) 3APCl.

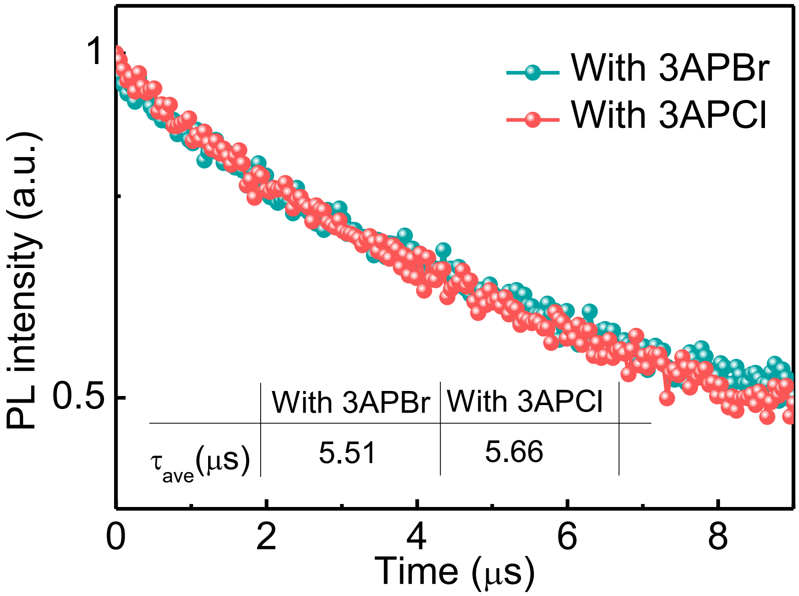

Supplementary Fig. 22| TRPL profiles of the perovskite films with 3APX (X = Br, Cl).

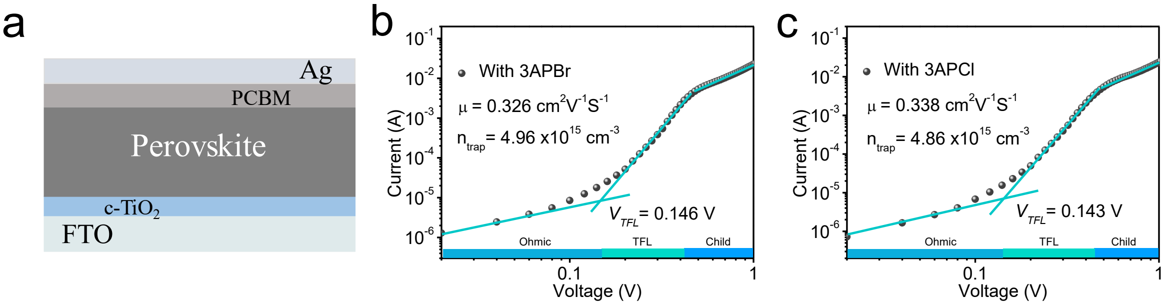

Supplementary Fig. 23| SCLC analyses of the fabricated devices. (a) Electron-only devices with the FTO/c-TiO_2/perovskite/PCBM/Ag architecture. Dark *J*–*V* curves of the devices with (b) 3APBr and (c) 3APCl.

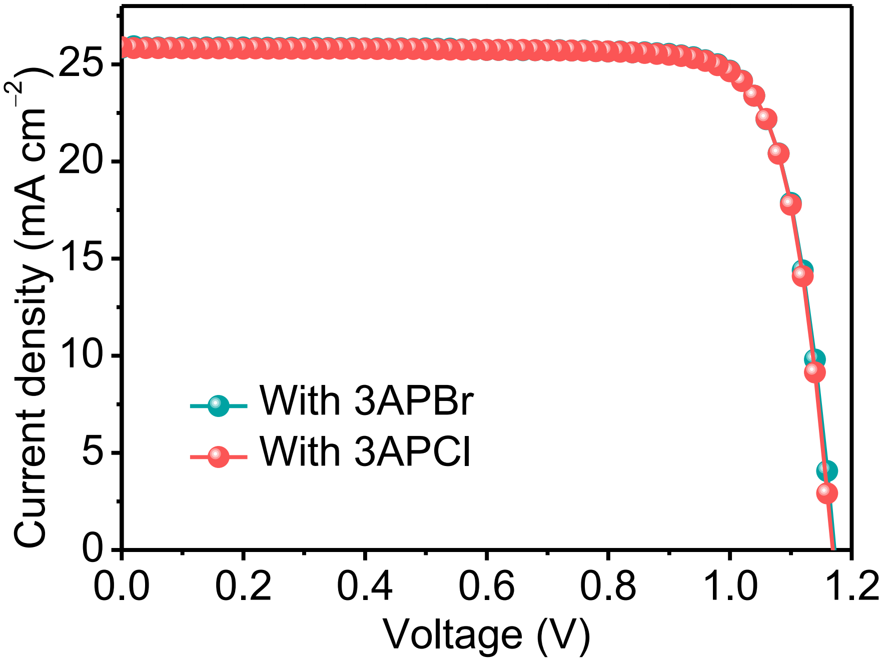

Supplementary Fig. 24| Current density–voltage (*J*–*V*) curves obtained for the devices with 3APX (X = Br, Cl).

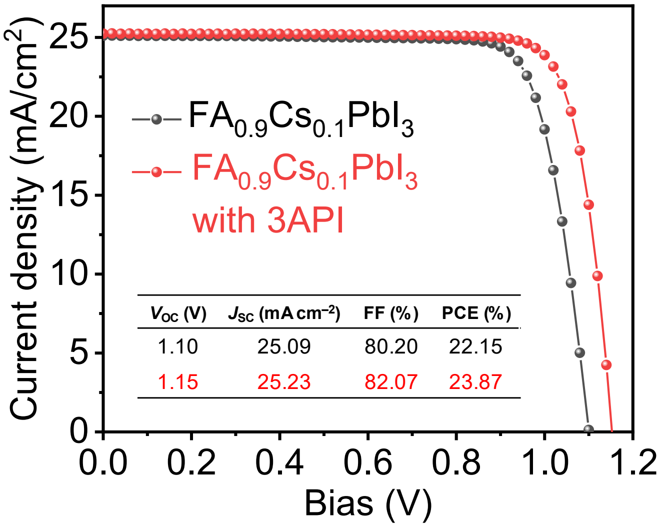

Supplementary Fig. 25| The J-V curves of $FA_{0.9}Cs_{0.1}PbI_3$ device with/without 3API.

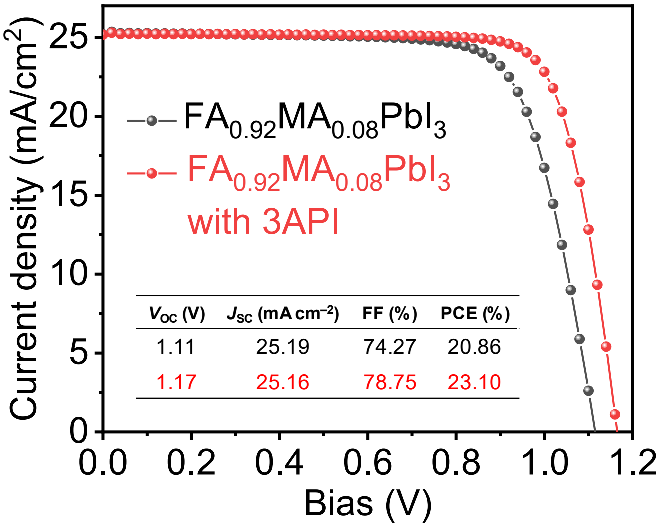

Supplementary Fig. 26| The J-V curves of $FA_{0.92}MA_{0.08}PbI_3$ device with/without 3API.

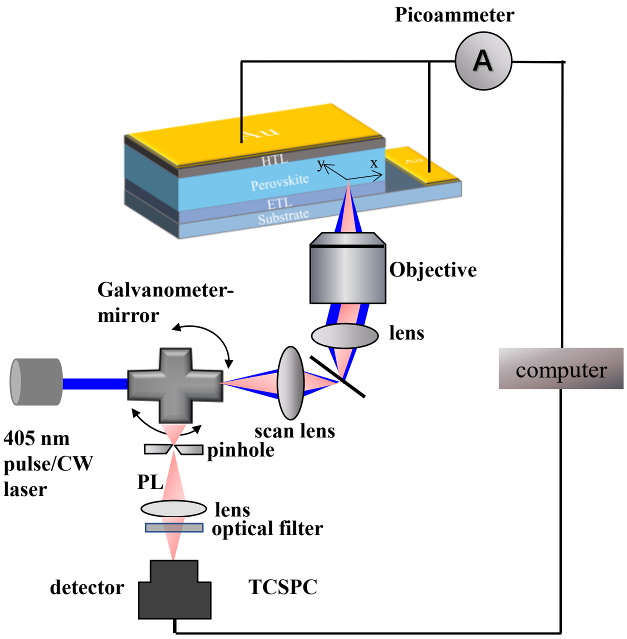

Supplementary Fig. 27| Schematic presentation of the laser-scanned and time-resolved PL microscopy coupled with a photocurrent detection module.

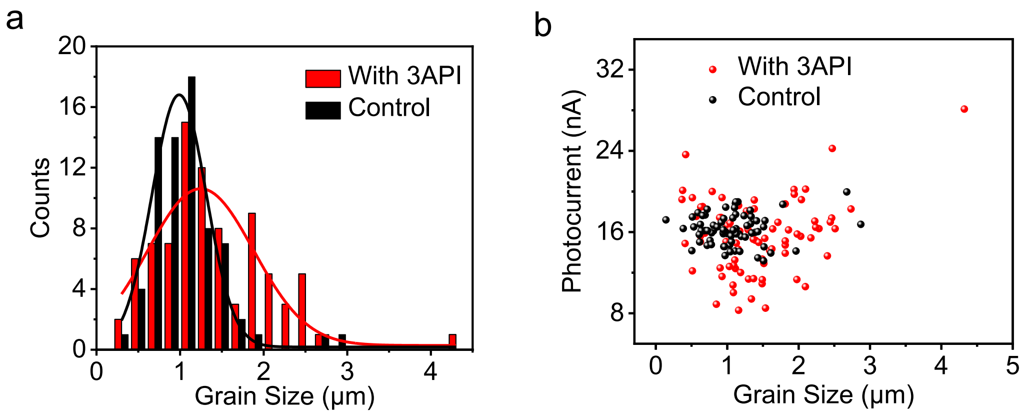

Supplementary Fig. 28| Statistical Analysis. (a) Histogram of grain size statistics extracted from PL intensity images. (b) Scatter plot of photocurrent statistics extracted from photocurrent images.

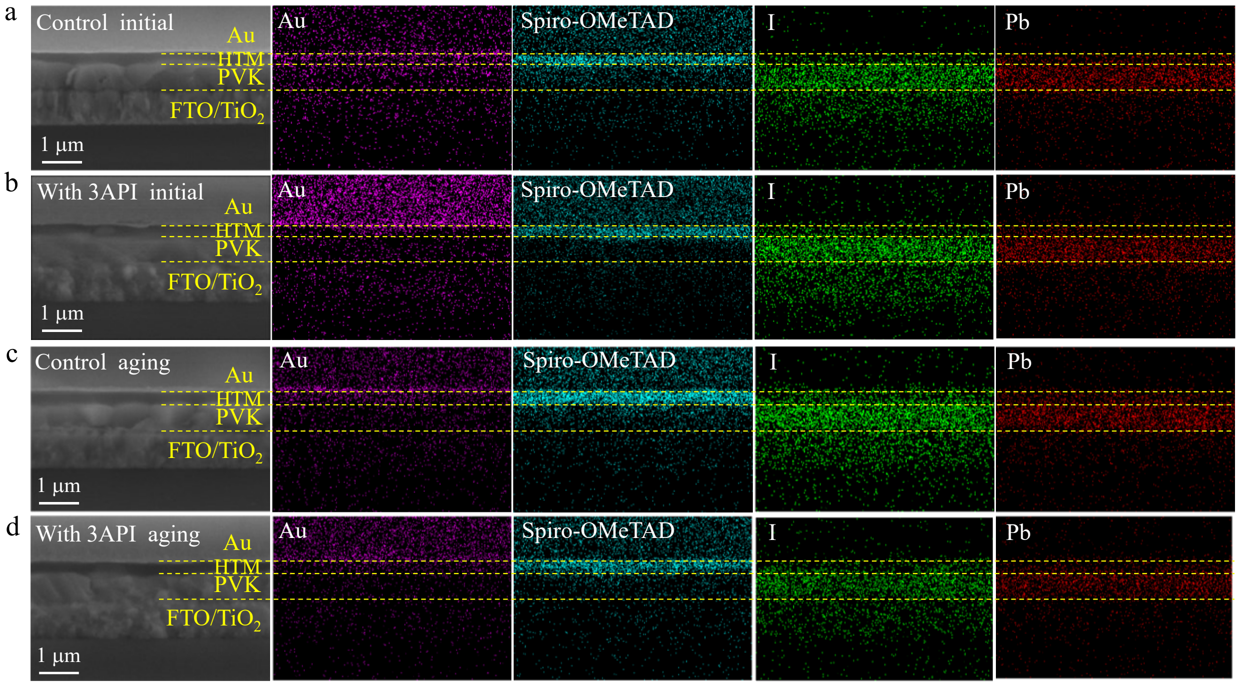

Supplementary Fig. 29| The cross-sectional SEM images and corresponding Energy Dispersive Spectroscopy (EDS) of the devices with or without 3API before (a, b, denoted as initial) and after aging (c, d), respectively. "HTM" represents the hole transport layer, which is the Spiro-OMeTAD layer, and "PVK" represents the perovskite layer, whose representative elements include "I" and "Pb".

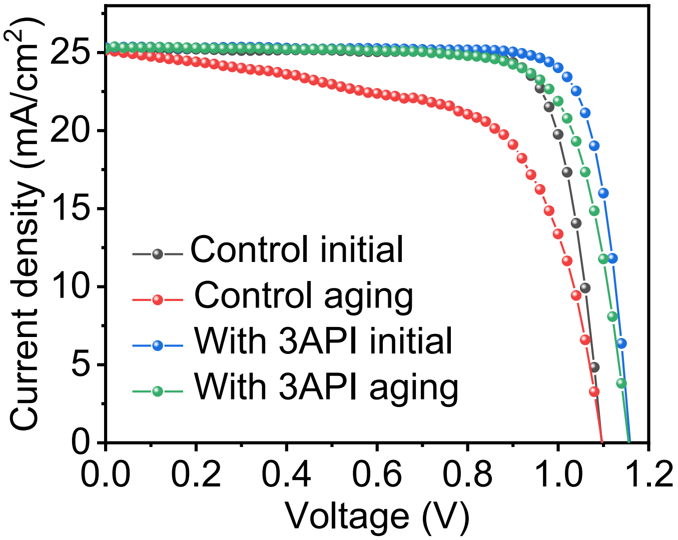

Supplementary Fig. 30| Initial and aging *J–V* curves for devices with long-term stability testing.

Supplementary Table 1| Structural parameters of the $(3AP)PbI_4$ single crystal.

	$(3AP)PbI_4$
Empirical formula	C6 H9 I4 N3 Pb
Formula weight	837.95
Temperature	190(2) K
Wavelength	1.34139 Å
Crystal system	Monoclinic
Space group	C2/c
Unit cell dimensions	a = 17.0883(11) Å, α = 90°<br>b = 9.4534(6) Å, β = 101.866(2)°<br>c = 19.6226(12) Å, γ = 90°
Volume	3102.1(3) $Å^3$
Z	8
Density (calculated)	3.588 g/cm^3
Absorption coefficient	57.068 mm^{-1}
Independent reflections	2759 [R_{int} = 0.0604]
Final R indices [I > 2σ(I)]	R_{obs} = 0.1413, wR_{obs} = 0.0.3501
R indices [all data]	R_{all} = 0.1443, wR_{all} = 0.3532
Largest diff. peak and hole	6.663 and -5.303 $e·Å^{-3}$

Supplementary Table 2| Interlayer distances of some molecular ligands.

	Interlayer distance (Å)
3AP	3.4534
3AMP	3.8573
3AMPY	3.7106
BA	5.4209
PEA	12.2104

Supplementary Table 3| Adsorption energies, I^- defect formation energies, and energy barriers of I^- diffusion calculated for the $FAPbI_3$ (001) (FAI-terminated) and $MAPbI_3$ (001) (MAI-terminated) systems with/without 3AMP, 3AMPY, 3AP, BA, PEA ligands.

Adsorption system	Adsorption energy (eV)	Defect formation energy (eV)	Energy barrier (eV)
$FAPbI_3$_001	–	1.422	0.737
$MAPbI_3$_001	–	1.075	0.688
$FAPbI_3$_3AMP	−2.784	3.574	0.907
$FAPbI_3$_3AMPY	−2.988	3.791	0.966
$FAPbI_3$_3AP	−3.135	3.812	1.467
$FAPbI_3$_BA	−2.968	3.716	1.293
$FAPbI_3$_PEA	−2.498	3.584	0.861

Supplementary Table 4| Perovskite photovoltaic parameters of the devices containing 0%, 4%, and 8% mol.% 3API.

		PCE (%)	V_{OC} (V)	J_{SC} (mA cm^{-2})	FF (%)
Control	max	22.76	1.123	24.94	81.30
	average	22.08±0.56	1.115±0.006	24.78±0.17	79.93±1.73
With 3API 4 mol %	max	25.29	1.181	26.04	82.21
	average	24.52±0.34	1.169±0.005	25.69±0.24	81.64±0.61
With 3API 8 mol %	max	23.84	1.157	25.47	80.92
	average	23.41±0.19	1.150±0.005	25.24±0.18	80.64±0.35

Supplementary Table 5| Parameter statistics of the PSCs with 3APX (X = Br, Cl).

		PCE (%)	V_{OC} (V)	J_{SC} (mA cm^{-2})	FF (%)
With 3APBr	max	24.66	1.172	25.85	81.41
	average	23.85±0.33	1.164±0.006	25.57±0.29	80.16±0.68
With 3APCl	max	24.63	1.169	25.87	81.44
	average	24.06±0.29	1.167±0.002	25.64±0.20	80.42±0.60

Supplementary Table 6| The photovoltaic parameters of the devices before and after aging.

		PCE (%)	V_{OC} (V)	J_{SC} (mA cm^{-2})	FF (%)
Control	Initial	22.11	1.096	25.24	79.91
	Aging	17.37	1.097	25.14	62.96
With 3API	Initial	24.02	1.158	25.32	81.86
	Aging	22.31	1.155	25.25	76.45
