# Supplementary material for: One-stone-for-two-birds strategy to attain beyond 25% perovskite solar cells
Source: Nat Commun. 2023 Feb 15;14:839. doi: 10.1038/s41467-023-36229-1 (PMC9932071; doi:10.1038/s41467-023-36229-1)
Supplement: Supplementary file 3 — Solar Cells Reporting Summary [file 41467_2023_36229_MOESM3_ESM.pdf]

nature research

Corresponding author(s): Wenming Tian
Shengzhong(Frank) Liu
Kui Zhao

Solar Cells Reporting Summary

Nature Research wishes to improve the reproducibility of the work that we publish. This form is intended for publication with all accepted papers reporting the characterization of photovoltaic devices and provides structure for consistency and transparency in reporting. Some list items might not apply to an individual manuscript, but all fields must be completed for clarity.

For further information on Nature Research policies, including our data availability policy, see Authors & Referees.

Experimental design

Please check: are the following details reported in the manuscript?

1. Dimensions

Item	Yes	No	Details
Area of the tested solar cells	☒	☐	see Methods
Method used to determine the device area	☒	☐	see Methods

2. Current-voltage characterization

Item	Yes	No	Details
Current density-voltage (J-V) plots in both forward and backward direction	☒	☐	see Methods and Figure 2c
Voltage scan conditions<br>*For instance: scan direction, speed, dwell times*	☒	☐	see Methods
Test environment<br>*For instance: characterization temperature, in air or in glove box*	☒	☐	see Methods
Protocol for preconditioning of the device before its characterization	☒	☐	see Methods
Stability of the J-V characteristic<br>*Verified with time evolution of the maximum power point or with the photocurrent at maximum power point; see ref. 7 for details.*	☒	☐	see Figure 2d

3. Hysteresis or any other unusual behaviour

Item	Yes	No	Details
Description of the unusual behaviour observed during the characterization	☒	☐	see Methods and Figure 2c
Related experimental data	☒	☐	see Figure 2c

4. Efficiency

Item	Yes	No	Details
External quantum efficiency (EQE) or incident photons to current efficiency (IPCE)	☒	☐	see Figure 2e
A comparison between the integrated response under the standard reference spectrum and the response measure under the simulator	☒	☐	see Methods
For tandem solar cells, the bias illumination and bias voltage used for each subcell	☐	☒	This is not a tandem solar cell

5. Calibration

Item	Yes	No	Details
Light source and reference cell or sensor used for the characterization	☒	☐	see Methods
Confirmation that the reference cell was calibrated and certified	☒	☐	see Methods

Calculation of spectral mismatch between the reference cell and the devices under test

☐ Yes ☒ No — We did not do the calculation of spectral mismatch.

6. Mask/aperture

Size of the mask/aperture used during testing

☒ Yes ☐ No — see Methods

Variation of the measured short-circuit current density with the mask/aperture area

☐ Yes ☒ No — We did not do the current variation test with different mask areas.

7. Performance certification

Identity of the independent certification laboratory that confirmed the photovoltaic performance

☒ Yes ☐ No — Some cells were sent to National Institute of Metrology (NIM, China) for certification.

A copy of any certificate(s)
Provide in Supplementary Information

☒ Yes ☐ No — see Supplementary Fig. 9

8. Statistics

Number of solar cells tested

☒ Yes ☐ No — see Figure 2b

Statistical analysis of the device performance

☒ Yes ☐ No — see Figure 2b

9. Long-term stability analysis

Type of analysis, bias conditions and environmental conditions
For instance: illumination type, temperature, atmosphere humidity, encapsulation method, preconditioning temperature

☒ Yes ☐ No — see Figure 5
